# Developmental Lead Exposure Alters Synaptogenesis through Inhibiting Canonical Wnt Pathway *In Vivo* and *In Vitro*

**DOI:** 10.1371/journal.pone.0101894

**Published:** 2014-07-07

**Authors:** Fan Hu, Li Xu, Zhi-Hua Liu, Meng-Meng Ge, Di-Yun Ruan, Hui-Li Wang

**Affiliations:** 1 School of Biotechnology and Food Engineering, Hefei University of Technology, Hefei, Anhui, China; 2 School of Life Sciences, University of Science and Technology of China, Hefei, Anhui, China; University of Texas Medical Branch, United States of America

## Abstract

Lead (Pb) exposure has been implicated in the impairment of synaptic plasticity in the developing hippocampus, but the mechanism remains unclear. Here, we investigated whether developmental lead exposure affects the dendritic spine formation through Wnt signaling pathway *in vivo* and *in vitro*. Sprague–Dawley rats were exposed to lead throughout the lactation period and Golgi-Cox staining method was used to examine the spine density of pyramidal neurons in the hippocampal CA1 area of rats. We found that lead exposure significantly decreased the spine density in both 14 and 21 days-old pups, accompanied by a significant age-dependent decline of the Wnt7a expression and stability of its downstream protein (β-catenin). Furthermore, in cultured hippocampal neurons, lead (0.1 and 1 µM lead acetate) significantly decreased the spine density in a dose-dependent manner. Exogenous Wnt7a application attenuated the decrease of spine density and increased the stability of the downstream molecules in Wnt signaling pathway. Together, our results suggest that lead has a negative impact on spine outgrowth in the developing hippocampus through altering the canonical Wnt pathway.

## Introduction

Lead (Pb) is an environmental neurotoxic metal, which has a well-accepted negative impact on children's cognitive development [1], [2]. It is also a potential high-risk factor for attention deficit hyperactivity disorder in children [3]–[5]. Developmental lead exposure has been found to decrease the induction and amplitude of long-term potentiation (LTP), the cellular model of learning and memory, in the rat hippocampus [6], [7]. Additionally, morphological analysis shows that there is an obvious reduction in the length of dendritic field and the number of dendritic branches of hippocampal dentate granule cells after developmental lead exposure [8], [9]. Previous studies have reported that developmental lead exposure causes alteration of NMDAR subunit ontogeny and disruption of its downstream signaling [10], [11], which are associated with deficits in hippocampal LTP [12]. Although alteration of dendritic branching of granule cells in the developing hippocampus after lead exposure has been reported, the outstanding question remains whether lead exposure affects synaptogenesis of pyramidal cells, which may also contribute to deficits in synaptic plasticity in the hippocampus.

Excitatory synapses, involving in the induction of LTP, are mostly located on dendritic spines, which are one of the main structural features of pyramidal neurons [13]. The appearance of new spines often coincides with synapse formation [14]. It has been found that several intracellular factors participate in the regulation of formation and maturation of dendritic spines throughout development [15], [16]. Extracellularly secreted molecules, such as Wnt, have also been shown to promote spine development and maturation [17]. Previous study has found that Wnt secreted proteins are broadly distributed in the nervous system, especially in the hippocampus [18]. These proteins play an important role in the nervous system maturation by regulating neuronal polarity, synapse formation, synaptic plasticity and transmitter release [19]. In cultured hippocampal neurons, Wnt5a mainly participates in postsynaptic development of both GABAergic and glutamatergic synapses [20], [21]. Wnt7a increases presynaptic protein clustering and modulates synaptic vesicle cycling through the canonical pathway (or Wnt/β-catenin pathway) [18]. Electrophysiological analysis of the adult rat hippocampal slices indicates that Wnt7a increases neurotransmitter release at CA3-CA1 synapses by increasing the frequency of miniature excitatory post-synaptic currents (mEPSC) [18]. It could also induce postsynaptic receptors expression through the non-canonical pathway [18], [22]. In the Wnt7a deficient mice, there are obvious defects in spine morphogenesis and mossy fiber-CA3 synaptic transmission, both of which are dependent on the post-synaptic non-canonical Wnt pathway (Ca^2+^/Calmodulin-dependent pathway) [17]. Since Wnt signaling is required for multiple aspects of synaptogenesis, we postulated that developmental lead exposure may disrupt Wnt signaling pathway, then leading to the damage to dendritic spine formation in hippocampal neurons.

The current study aims to explore whether lead exposure alters dendritic spine morphology of pyramidal neurons in the hippocampus of developing rats. Wnt7a and its downstream molecular expression were also determined to examine whether the morphological alteration of dendritical spine after lead exposure involving the Wnt signaling pathway.

## Materials and Methods

### Experimental animals and tissue collection

Sprague–Dawley (SD) rats were obtained from the Laboratory Animal Center, Anhui Medical University, China. All animal experiments were performed following the guidelines of the National Institutes of Health Guide for the Care and Use of Laboratory Animals, and were approved by the Institutional Animal Care and Use Committee of Hefei University of Technology, China. The method for chronic lead exposure was referred to the previous studies [23], [24]. SD rat dams were randomly divided into two groups: control and lead-exposed group, drinking distilled water and lead water (250 ppm lead acetate in distilled water, 30 ml/day), respectively. The lead-exposed pups acquired lead via milk of dams during lactation period. *In vivo* experiments were carried out at the age of 14 and 21 days in a total of 18/19 rats (mean weight, 11.11±0.59 g and 10.7±0.54 g, p>0.05) and 20/20 rats (mean weight, 16.8±0.91 g and 17.26±0.85 g, p>0.05) for the control and lead-exposed groups, respectively.

After lead treatment, rat pups were deeply anesthetized with CO_2_ and decapitated. Brains were then removed from the skull quickly within 1 min. Some brains were longitudinally cut into two halves; the left hemibrain was frozen and stored at −80°C for lead concentration assay, while the right part for morphological staining. The other brains were used for examining special protein expression.

### Lead concentration determination in hippocampus

For lead concentration assay, the tissue sample of hippocampus (<0.5 g) was added with nitric acid (excellent pure GR, 4 ml) and 30% hydrogen peroxide (AR, 2 ml) in nitrolysis tube overnight at room temperature, then nitrolyzed for 30 min in the microwave nitrate pyrolysis furnace (MARSXpress, CEM Corporation, USA). Lastly, the lead concentration within sample (without any solids) was detected by the graphite furnace atomic spectrophotometry (The PerkinElmer AAnalyst 800, USA).

### Golgi-Cox staining, neuron-selection criteria and spine density assay

The brain was processed by Golgi-Cox staining method, which is a well-known method used to staining whole neuron dendrites and spines *in vivo* [25]–[27]. In brief, the brains were first stored in the dark for two days (37°C) in Golgi-Cox solution, and then were sectioned at a thickness of 200 µm in the coronal plane with a vibratome (VT1000S, Leica, Germany). The coronal sections containing hippocampal CA1 area (Bregma, −2.2∼−6.2 mm) were used in the present study. We collected one out of every 3 sections and got about 5 sections per animal. Those sections were mounted to 2% gelatin-coated slides and stained with ammonia for 60 min, washed with water three times, followed by Kodak Film Fix for 20 min, and then washed with water, dehydrated, cleared, and mounted using a resinous medium. At last, pyramidal neurons in hippocampus (CA1) were imaged with a Nikon widefield microscope (Eclipse 80i, Nikon, Japan) by using a 40× objective. From all sections in each rat, about 6 neurons per section were chosen. There were five main criteria used to select pyramidal neurons in hippocampus for photographing and analysis: (1) location of the cell soma in hippocampal CA1 area, (2) triangular soma, (3) presence of an apical dendritic shaft and at least three primary basilar dendritic shafts, each of which branched at least once, (4) full impregnation of neurons, (5) no morphological changes attributed to Golgi-Cox staining.

Then, spine density (spine number per 10 µm) for each neuron was analyzed by using MATLAB software [27]. The spines counted in the present study were on 2∼3 stretches of the secondary dendrite about 20 µm in length.

### Neuronal cultures

Primary hippocampal cultures were prepared from brains of SD rats at postnatal day 0 (P0) [28]. In briefly, hippocampus (without DG region) were dissected and dissociated with trypsin (0.03%) for 19 minutes, triturated with decreasing sizes of fire-polished pipettes. Cultures were then plated (100 cells per mm^2^) on dishes precoated with poly-L-lysine (0.5 mg/ml) (Sigma-Aldrich, USA). The next day, culture media was 70% replaced with serum-free neurobasal media supplemented with B27 and glutamax (GIBCO-Brl, Grand Island, NY). Then on the 7 days in vitro (DIV7), half the media was renewed with fresh neurobasal media, added with Ara-C (2 µl/ml from 4 mM stock, Sigma-Aldrich, USA) to inhibit the glial cells proliferation. For lead exposure, cultures were treated with lead acetate (0.1 and 1 µM, Sigma-Aldrich, USA) for 5 days from DIV 7 to DIV 12 with or without recombinant Wnt7a (100 ng/ml, R&D system, USA) [17]. Stock solutions of lead acetate (100 µM, 1 mM) were made and kept at 4°C and were diluted to working concentration (0.1 µM and 1 µM) [11] with feeding medium before use.

### Lentivirus production-enhanced GFP (EGFP) expression

Lentiviral vectors for enhanced GFP (EGFP) gene expression was a kind gift of Professor Guo-Qiang Bi (University of Sciences and Technology of China, Anhui, China), which were produced in human embryonic kidney 293FT cells by using the 2nd lentivirus vector generation packaging system.

Hippocampal cultures were lentiviral infected with the EGFP (as a morphological indicator for analysis of primary cultured neurons [26], [29]) at DIV 6 and fixed with 4% paraformaldehyde (PFA) (15 min, room temperature) at DIV 12 after 5 days lead exposure (0.1 µM and 1 µM) with or without 16 hours Wnt7a treatment (100 ng/ml). For morphological analysis, cells were imaged with an Olympus FV1000 BX61WI laser-scanning confocal microscope, 0.5 µm z step, at constant laser intensity and photomultiplier tube settings (settled by the control group). The morphological criteria and the analysis of spine density within those EGFP labeled pyramidal neurons were same with that in the Golgi-Cox staining neurons assay.

### Western blotting assay

The hippocampal protein (CA1 area) from pups at the age of 14 and 21 days was obtained from hippocampus by using homogenizer for 1 minute and lysis for 1 hour on ice. The protein concentration was determined by using the Bicinchoninic Acid (BCA) method. The protein from hippocampus culture was directly harvested after lead treatment with or without Wnt7a by SDS-PAGE sample buffer solution. An equal amount of samples was resolved by an 8.5% SDS-PAGE gel. Resolved proteins were transferred to a PVDF membrane. The non-specific sites were blocked with 5% non-fat dry milk, followed by incubation with primary antibodies (β-actin and Wnt7a were purchased from Abcam, β-catenine and phospho-β-catenine were purchased from Cell Signaling Technology, USA). The membranes were washed three times and incubated with the appropriate horseradish peroxidase-conjugated secondary antibody to detect bands by enhanced chemiluminescence (ECL, GE Healthcare). The bands developed on the films were quantified by a densitometer. All results were normalized against β-actin.

### Statistical analysis

The statistical differences between groups were analyzed using unpaired-T test or One-way ANOVA followed by a Bonferroni post-hoc test. Data were expressed as mean ± S.E.M. P<0.05 was considered as the statistical difference.

## Results

### Tissue lead accumulation after chronic lead exposure *in vivo*

**Figure 1:**
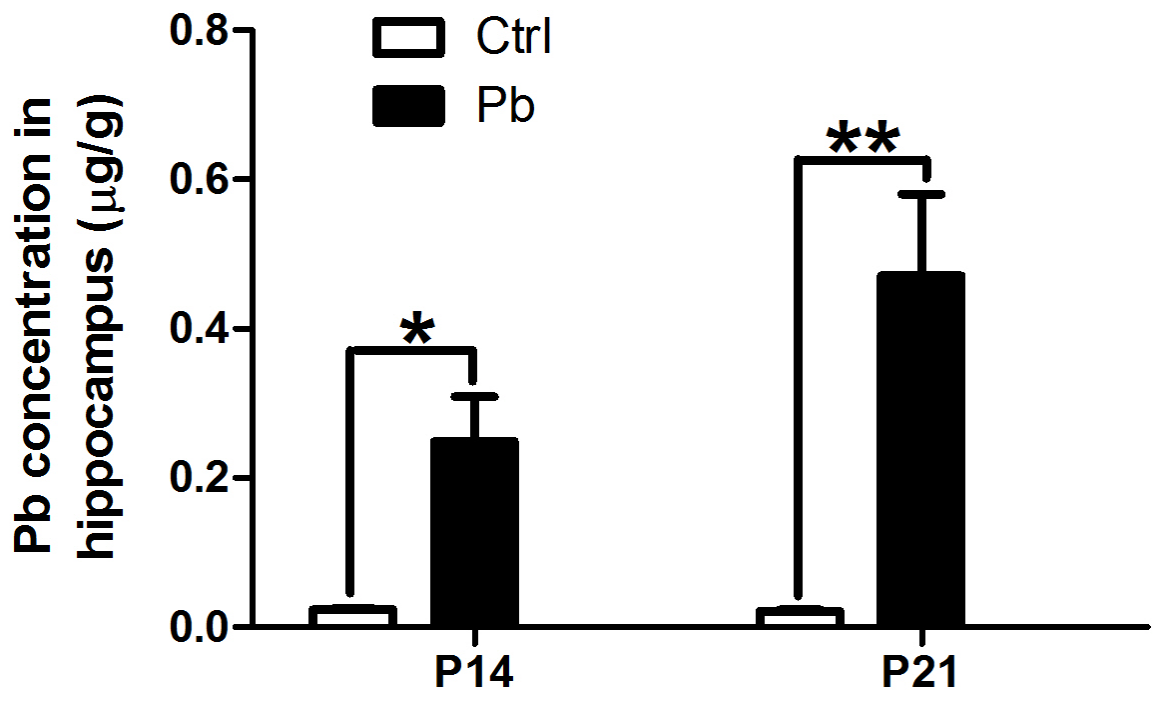
Lead accumulations in hippocampus in the control and chronic lead-exposed rats. The lead levels in P14 and P21 rats with or without lead exposure were determined. Histograms were plotted by the mean of 8 rat hippocampus per group. (*p<0.05, **p<0.01).

To examine lead accumulation within the hippocampus, tissue lead concentration was determined with graphite furnace atomic spectrophotometry. In the P14 rats, lead concentration in the hippocampus was 0.023±0.002 µg/g in control animals, while 0.249±0.06 µg/g in lead exposed animals. An additional 7 days lead exposure increased the concentration even further to 0.471±0.11 µg/g. In the age-matched control group, this value was 0.021±0.002 µg/g. Thus, lead exposure obviously increased lead levels within the hippocampus (Fig. 1, n = 8, P<0.05 and P<0.01, respectively).

### Lead-exposure induced changes of spine density and Wnt pathway *in vivo*

**Figure 2:**
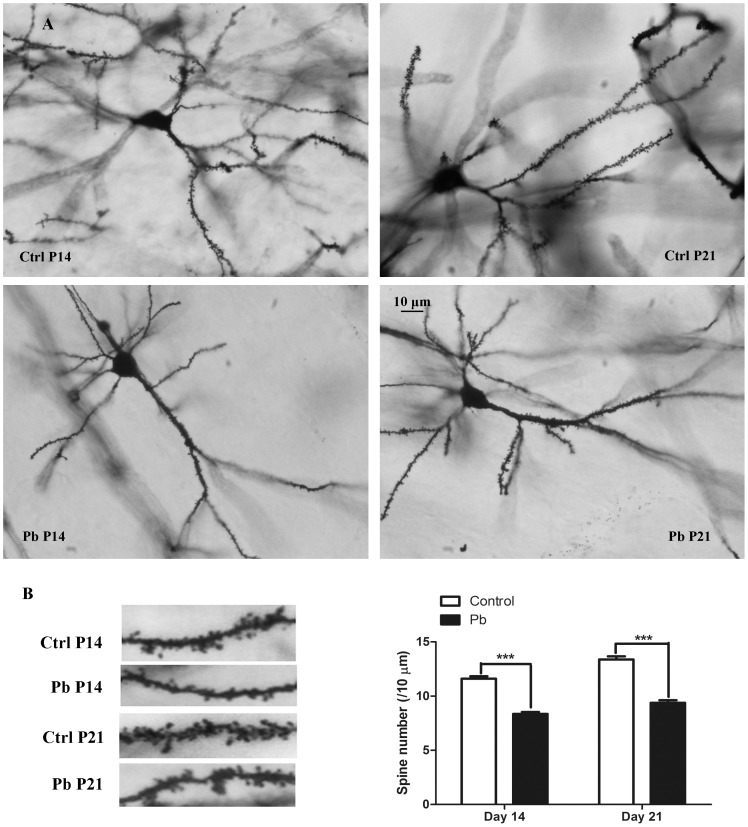
The dendritic spine alteration of pyramidal neurons in the hippocampus of P14 and P21 rats after chronic lead exposure. A) Golgi-Cox impregnated dendritic arborization and dendritic spines in four groups: control (Ctrl) P14, lead (Pb) P14, Ctrl P21, lead (Pb) P21. Scale bar = 10 µm; B) Representative sections (20 µm) of Golgi-Cox stained dendrites of pyramidal neurons in hippocampus in four groups; C) Histograms plot showing the alteration of dendritic spine density (spines/10 µm) after lead exposure in P14 and P21 rats. (***p<0.001).

Exposure to lead in rats was initiated at embryonic phase and terminated at postnatal 21 (P21) days. This time period was considered as the critical window for rodent nervous system development [30], and allowed us to examine the effects of lead on the synapse formation. By Golgi-Cox staining method, the P14 and P21 poisoned rats exhibited 32.83% and 24.11% decrease in the number of dendritic spine on hippocampal CA1 area, respectively (P<0.001, Fig. 2). This result showed that lead exposure significantly impaired spine formation in developmental hippocampus.

**Figure 3:**
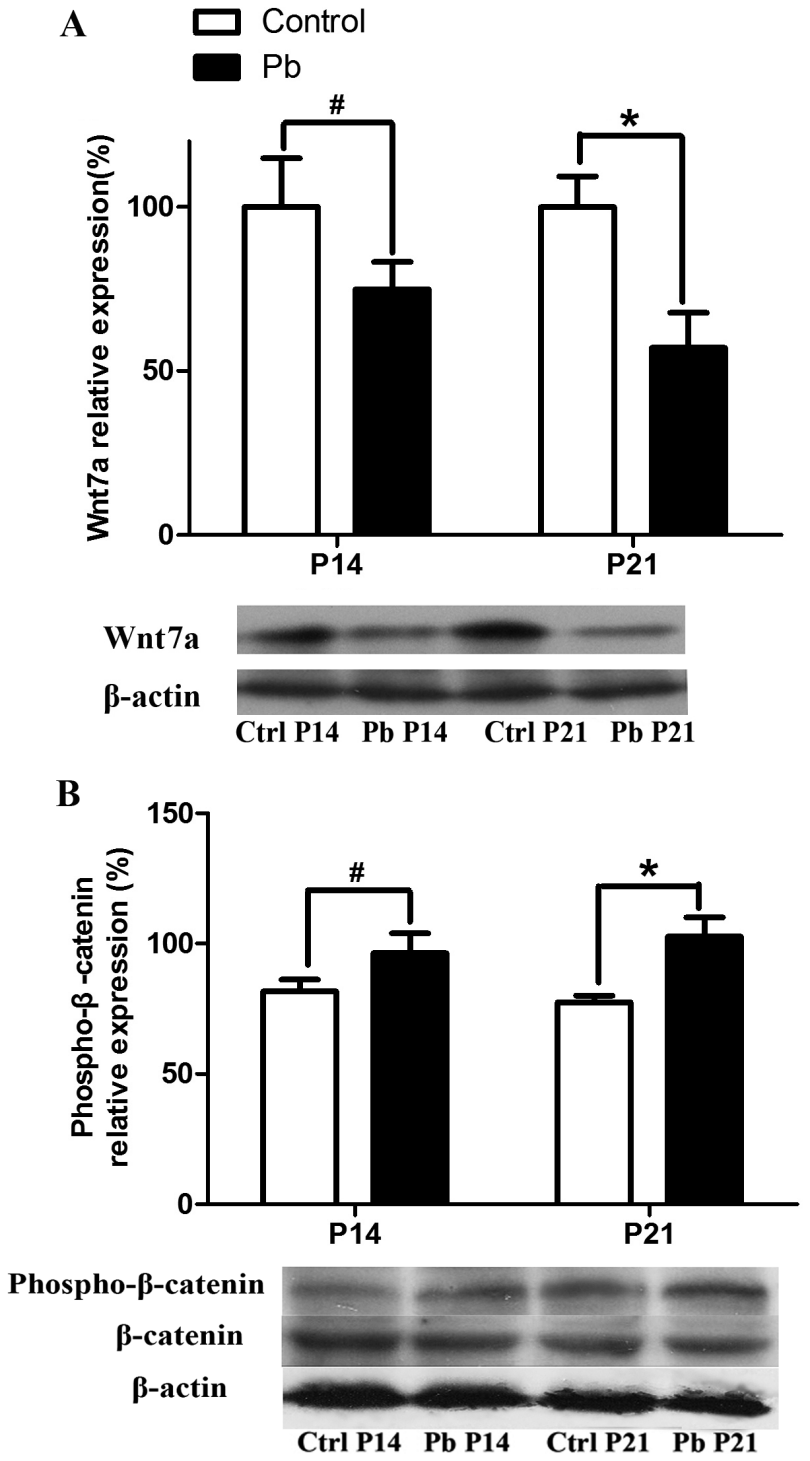
Effect of developmental lead exposure on Wnt/β-catenin pathway in vivo. A) Representative immunoblot and corresponding densitometric analysis showed expression of Wnt7a which was normalized to β-actin in the control and lead-treated groups in P14 and P21 rats. (#p>0.05 and *p<0.05). B) Representative immunoblot and corresponding densitometric analysis showed the ratio of expression of phosphorylated β-catenin (phospho-β-catenin) to total β-catenin in control and lead treated groups in P14 and P21 rats. (#p>0.05 and *p<0.05).

Given the broad effects of Wnt secreted proteins on synapse formation and synaptic strength, we asked whether lead-induced spine deficit is resulted from the alteration of Wnt signaling. Wnt7a expression in hippocampal CA1 area after chronic lead exposure was examined by Western blotting assay. As shown in Fig. 3A, the relative amount of Wnt7a in P14 and P21 of lead-exposed rats were downregulated to 74.81% and 57.06% as compared to control group (p>0.05 and p<0.05, respectively). It suggests that the decrease of Wnt7a expression showed an age-dependent decline manner in lead-induced synapse damage in developmental rats.

In the nervous system, synapse formation involves the accumulation of cytosolic β-catenin, which could enter into nucleus to active the target gene transcription [31]. The absent of Wnt proteins causes β-catenin ubiquitination and subsequent proteasomes-dependent degradation by increasing the phosphorylation level of β-catenin. To further determine the effect of lead on the activity of canonical Wnt pathway (Wnt/β-catenin pathway), we examined phosphorylation of β-catenin using Western blotting assay. As shown in Fig. 3B, lead increased the expression of phosphorylated β-catenin about 18.00% and 29.92% in P14 and P21 rats (P>0.05 and p<0.05, respectively). It was consistent with the decreasing of Wnt7a expression, which showed the impairment of the activity of Wnt/β-catenin pathway after developmental lead exposure.

### Wnt7a rescues changes of spine density and Wnt pathway after lead treatment *in vitro*

**Figure 4:**
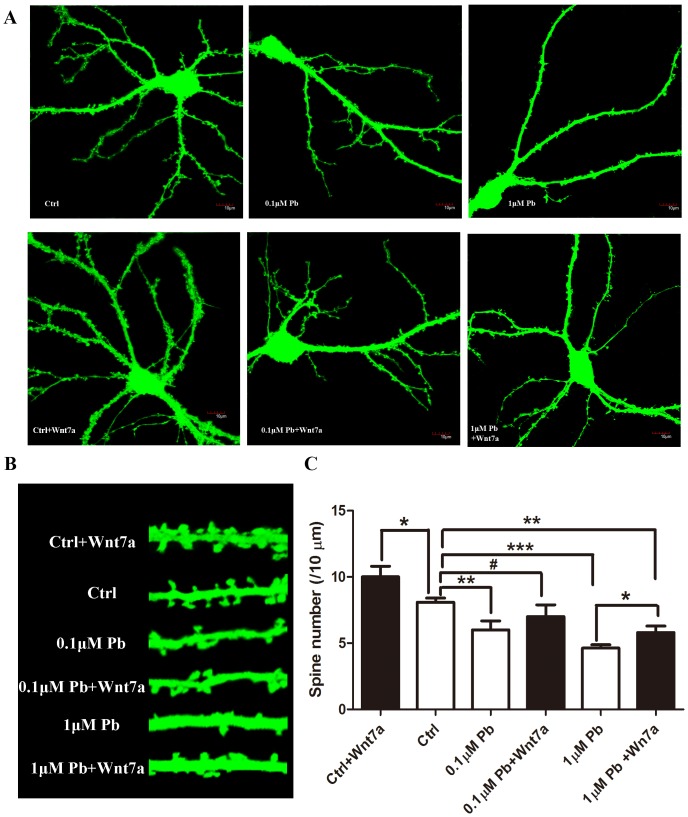
Effect of Wnt/β-catenin pathway on the dendritic spine density of hippocampal pyramidal neurons after lead treatment *in vitro*. A) Representative EGFP-transfected hippocampal neurons in the control group (Ctrl), the lead-treated groups (0.1 and 1 µM from DIV7 to DIV12), and the lead-treated groups with 16 hours Wnt7a treatment in DIV12 (0.1 µM Pb, 0.1 µM Pb+Wnt7a, 1 µM Pb, 1 µM Pb+Wnt7a), Scale bar = 10 µm; B) Representative sections (20 µm) of dendritic spines in four groups; C) Histograms plot showing the alteration of dendritic spine density (spines/10 µm) after lead treatment with or without Wnt7a. (*p<0.05, **p<0.01, ***p<0.001 and **#**p>0.05). All experiments were performed by using three independent cultures.

**Figure 5:**
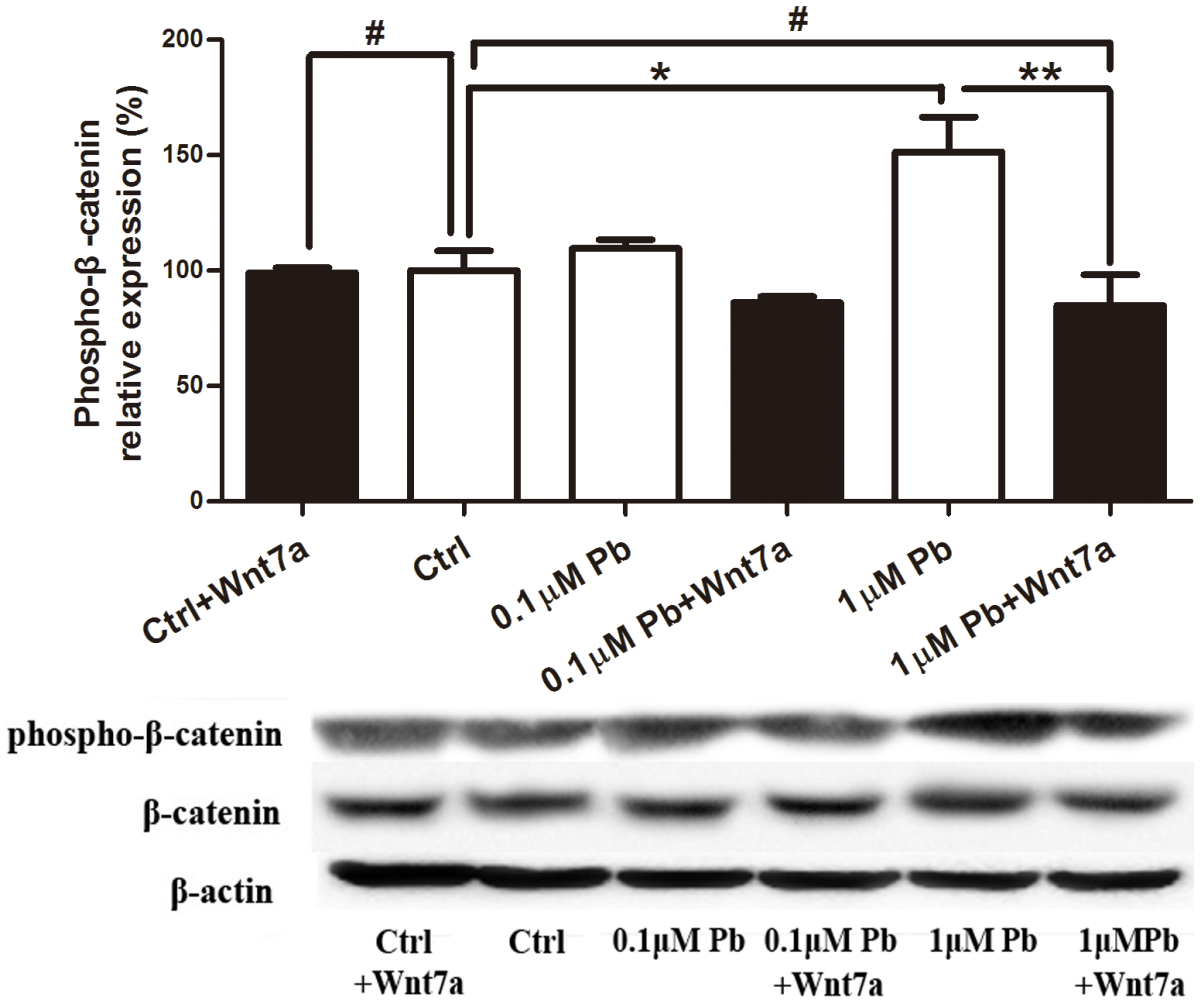
Effect of lead on the β-catenin phosphorylation *in vitro* and effect of Wnt7a on the lead-induced change of the β-catenin phosphorylation. Representative immunoblot and corresponding densitometric analysis showed the ratio of expression of phosphorylated β-catenin to total β-catenin in cultured hippocampal neurons of five groups: control (Ctrl), 0.1 µM lead (0.1 µM Pb), 0.1 µM lead with Wnt7a (100 ng/ml) (0.1 µM Pb+Wnt7a), 1 µM lead (1 µM Pb) and 1 µM lead with Wnt7a (100 ng/ml) (1 µM Pb+Wnt7a). (*p<0.05, **p<0.01, ***p<0.01 and **#**p>0.05). All experiments *in vitro* were performed by using three independent cultures.

In cultured hippocampal neurons, the lead (0.1 µM and 1 µM lead acetate) treatment was started at DIV 7 and ended at DIV 12. This time period corresponds to the peak of the synaptogenesis period in these cultures [32]. Analysis of hippocampal neurons, which were lentiviral transfected with EGFP, revealed an obvious decrease in spine density in a dose-dependent manner compared with the control group (Fig. 4A, 4B). Specifically, in 0.1 µM and 1 µM lead-treated group, the dendritic spine density was significantly decreased about 25.84% and 42.70%, compared to the control group (P<0.01 and P<0.001, respectively, Fig. 4C). By western blotting assay, 1 µM lead increased the phosphorylation level of β-catenin to 151.45% of the control level (P<0.05, Fig. 5).

It is well known that Wnt7a signaling is required in axons to regulate synapse formation [33]. To determine whether lead exposure affects spine formation through Wnt7a pathway, exogenous Wnt7a was added into the culture medium to examine the change of dendritic spine density. After Wnt7a application, spine density in 0.1 µM lead-treated group significantly increased to 86.53% of control non-exposed level (P>0.05, Fig. 4C). In 1 µM lead-treated group, Wnt7a also significantly inhibited the decrease of spine density (P<0.05), which was recovered to 71.69% of control level (Fig. 4C). Additionally, the phosphorylation level of β-catenin was decreased about 49.65% compared to 1 µM lead-treated group and almost same with the control group (P<0.01 and P>0.05, Fig. 5). These results demonstrate that the Wnt/β-catenin pathway is critical for the early structural effects of lead during synaptogenesis.

## Discussion

In this study, several important observations were obtained: 1) We found that developmental lead exposure could induce a decrease in the spine density of pyramidal neurons in the developmental hippocampus *in vivo*, observed as early as in P14 rats. 2) We demonstrated that canonical Wnt7a pathway is involved in the lead-induced hippocampus damage in rats. After chronic lead exposure, there was a significant decrease of Wnt7a expression, which was accompanied by an increase in phosphorylation of β-catenin, the key downstream component of canonical Wnt pathway. 3) In cultured hippocampal neurons, low level lead (0.1 and 1 µM) exposures caused a decrease in spine density in a dose-dependent manner. Exogenous Wnt7a application rescued lead-induced decrease of spine density, accompanied by an increase in β-catenin stability.

The nervous system is sensitive to environmental insults in the embryonic and early developmental periods, during which many important developmental processes occur, such as neuron proliferation, differentiation, migration and synaptogenesis [30]. In the present study, our results from *in vivo* experiments demonstrate that there was a significant increase of lead accumulation in the hippocampus after chronic lead exposure. Compared to the control group, lead concentration increased about 10 and 20 times in P14 and P21 lead-exposed rats, respectively. This observation is consistent with previous studies [3], [34]. It also confirms that lead could cross the brain-blood-barrier, and thus is toxic to the central neuron system (CNS). Additionally, our results show that lead exposure significantly decreased the spine density of pyramidal neurons in CA1 region of hippocampus. This spine density phenotype in the hippocampus became more severe with prolonged lead exposure, which is consistent with the change of lead accumulation. Our study demonstrates for the first time that lead-induced decrease of spine formation can be oberved as early as postnatal day 14, earlier than what has been reported in other group's study [8]. It is well accepted that excitatory synaptic inputs are mainly located on dendritic spines, which are small protrusions from dendritic shaft of neurons with a biochemically compartmentalized head. Accordingly, the decrease of spine density after developmental lead exposure also is accompanied by the decrease of the synapse formation. Moreover, synaptic plasticity has been associated with morphological changes of spines [35]. For example, LTP induces a significant increase in new spine formation [36], which is mediated by clustering and remodeling of spine F-actin [37]. *In vivo* studies using two-photon microscopy have also demonstrated spine morphological changes as a result of synaptic plasticity [36], [38]. We have previously shown that lead exposure impairs LTP induction in CA3-CA1 hippocampal synapses in 23∼30-day-old rats [39]. Our current study provides further structural evidence, the decrease of dendritic spine density in hippocampal CA1 area, which maybe involve the impairment of excitatory synape. To some extent, it explains the reduction of LTP amplitude for lead treatment in our previous reports.

In the neuronal culture system, high level lead exposure (10∼100 µM) could cause hippocampal neurons death [40]–[42] and low level also could induce decrease of NMDA receptor expression to inhibit the synapse development [11]. The present results firstly show that low concentrations of lead (as low as 0.1 µM) also can significantly lead to the morphological damage of hippocampal neurons during synaptogenesis. Our previous report reveals that lead causes a decrease in the frequency of spontaneous EPSC in hippocampal slices [43], which may involve the alteration of presynaptic transmitter release. Consistent with the potential presynaptic effect, lead was found to directly inhibit nicotinic acetylcholine receptor (nAChR) function and regulate presynaptic transmitter release in the hippocampus [44]. nAChR agonist, nicotine, can facilitate LTP in the hippocampus (CA1) to overcome the functional deficit in lead-exposed rats [39]. Additionally, lead exposure in cultured hippocampal neurons could cause a loss of synaptophysin and synaptobrevin to regulate presynaptic vesicular release [45]. Taken together, these results indicate that lead may reduce transmitter release by acting directly on the presynaptic site. It has been shown that the spontaneously glutamate release activates nearby spines, which could then lead to the rapid formation of spine head protrusions connecting to a presynaptic site [46]. It means that active neurotransmitter release is instrumental for synapse and dendritic spine formation. Thus, lead could cause spine density decrease through inhibiting presynaptic release.

Wnt family of secreted glycolipoproteins directs cell proliferation, cell polarity and cell fate determination during embryonic development and tissue homeostasis [47]. In the nervous system, Wnt signaling pathway plays an important role in regulating synapse contact through directing axon guidance, dendritic arborization, axonal remodeling and synapse formation [19]. Dysfunction of Wnt pathway is associated with several neuron system diseases, such as, schizophrenia and Alzheimer's diseases (AD) [48], [49]. Wnt7a, a kind of Wnt components, is abundant and broadly distributed in the hippocampus. It regulates synaptic vesicle cycling of hippocampal neurons through a mechanism requiring Dvl1 and activation of the canonical Wnt pathway in axons [18], [50]. Some studies also show that Wnt7a regulates glutamate release probability [18], [33]. In the present study, developmental lead exposure caused a decrease in Wnt7a expression within hippocampal CA1 area, likely contributing to reduction of transmitter release and subsequent down-regulation of spine density in pyramidal neurons. After exogenous Wnt7a application, the decrease of dendritic spine density in lead treated hippocampal neurons were reversed, which was accompanied by the increase of Wnt signaling activity. The previous study has shown that developmental lead exposure is a contributing factor to the development of AD for lead-induced accumulation of amyloid-beta peptide [51], which could cause the loss of neuron synapse [48]. Dickkopf-1 (Dkk1), an antagonist of Wnt components, has been reported to decrease the synapse number of hippocampal neurons and participate in the amyloid-induced reduction in the synapse number [48]. Loss of Dkk1 in the adult rat brain also could induce the increase of neuronal dendritic complexity [52]. It suggests that change of Dkk1 expression may also play a key role in the lead-induced alteration of dendritic spine formation. Additionally, secreted frizzled-related protein 3, a naturally secreted Wnt inhibitor, could inhibit the Wnt pathway activity to block dendritic spine formation [53]. In our future study, we will further check the expression of these negative regulators in the dysfunction of Wnt pathway in lead-exposed hippocampus.

β-catenin is the centre molecule of the canonical Wnt pathway [31]. It is a transcriptional activator for the TCF/LEF-1 (T cell factor/lymphoid enhancer factor1) family of DNA binding proteins in the Wnt pathway [31]. In the absence of Wnt ligands, β-catenin is phosphorylated by glycogen synthase kinase 3β (GSK-3β) and is ubiquitylated and targeted for proteasome degradation [54]. Consistent with observed decrease in Wnt7a expression, phosphorylation of β-catenin was found significantly increased in the lead-treated group, further suggesting reduced activity of the canonical Wnt signaling pathway. Indeed, in cultured hippocampal neurons where we reproduced lead effect on spine density *in vivo*, exogenous Wnt7a application reversed the increase in β-catenin phosphorylation and rescued the decrease of spine density. This result further supports the notion that lead targets Wnt7a signaling pathway and impairs synaptogenesis of hippocampal neurons. It also has been reported that GSK-3β is an important regulator for development of AD [55], [56], which is a product of developmental lead exposure [51]. In the future study, we will examine the expression of GSK-3β to confirm the alteration of Wnt pathway activity for lead exposure in developmental hippocampus.

In conclusion, to our knowledge, this is the first study reporting the impact of lead exposure *in vivo* and *in vitro* on spine density in hippocampus. This morphological alteration was followed by the decrease of Wnt7a expression and the increase of phosphorylation of its downstream component, β-catenin. It suggested that there is a decrease of canonical Wnt pathway activity in the lead-induced neurotoxicity. Our results indicate that Wnt signaling pathway plays an important role in lead-induced alteration of spine formation. It might be a potential therapeutic target for lead-induced CNS damage during synaptogenesis.
